# Virus variation resources at the National Center for Biotechnology Information: dengue virus

**DOI:** 10.1186/1471-2180-9-65

**Published:** 2009-04-02

**Authors:** Wolfgang Resch, Leonid Zaslavsky, Boris Kiryutin, Michael Rozanov, Yiming Bao, Tatiana A Tatusova

**Affiliations:** 1 National Center for Biotechnology Information, National Library of Medicine, National Institutes of Health, Bethesda, Maryland, 20894, USA; 2 Department of Medical Microbiology, Center of Infectious Diseases, Leiden University Medical Center, PO Box 9600, E4-P 2300, RC Leiden, the Netherlands

## Abstract

**Background:**

There is an increasing number of complete and incomplete virus genome sequences available in public databases. This large body of sequence data harbors information about epidemiology, phylogeny, and virulence. Several specialized databases, such as the NCBI Influenza Virus Resource or the Los Alamos HIV database, offer sophisticated query interfaces along with integrated exploratory data analysis tools for individual virus species to facilitate extracting this information. Thus far, there has not been a comprehensive database for dengue virus, a significant public health threat.

**Results:**

We have created an integrated web resource for dengue virus. The technology developed for the NCBI Influenza Virus Resource has been extended to process non-segmented dengue virus genomes. In order to allow efficient processing of the dengue genome, which is large in comparison with individual influenza segments, we developed an offline pre-alignment procedure which generates a multiple sequence alignment of all dengue sequences. The pre-calculated alignment is then used to rapidly create alignments of sequence subsets in response to user queries. This improvement in technology will also facilitate the incorporation of additional virus species in the future. The set of virus-specific databases at NCBI, which will be referred to as Virus Variation Resources (VVR), allow users to build complex queries against virus-specific databases and then apply exploratory data analysis tools to the results. The metadata is automatically collected where possible, and extended with data extracted from the literature.

**Conclusion:**

The NCBI Dengue Virus Resource integrates dengue sequence information with relevant metadata (sample collection time and location, disease severity, serotype, sequenced genome region) and facilitates retrieval and preliminary analysis of dengue sequences using integrated web analysis and visualization tools.

## Background

The National Center for Biotechnology Information (NCBI) Virus Variation Resources (VVR) provide web retrieval interfaces, analysis and visualization tools for virus sequence datasets. In this paper we describe the recent extension of the collection of resources to include the Dengue Virus Resource in addition to the existing Influenza Virus Resource [1,2]. The NCBI Dengue Virus Resource was created to support a collaborative effort by the National Institute of Allergy and Infectious Diseases (NIAID), the Broad Institute, and the Novartis Institute for Tropical Diseases (NITD) to create a large collection of complete dengue genome sequences and provide access to the sequences and linked geographic and clinical information. This effort includes the NIAID-funded sequencing of dengue genomes from a wide geographic range by the Broad Institute and its collaborators.

**Figure 1:**
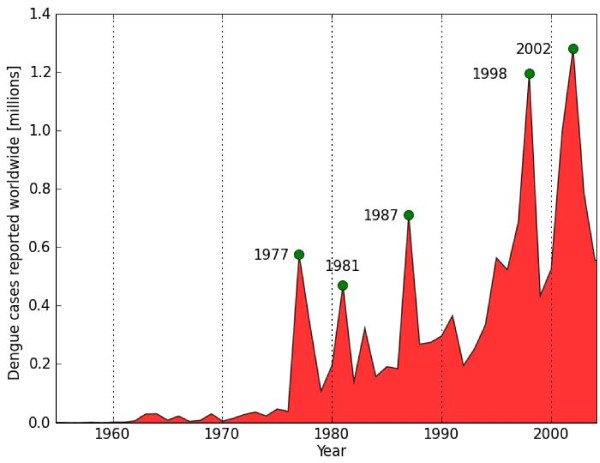
**Dengue cases reported worldwide from 1955 to 2004**. The number of dengue cases as reported in the WHO DengueNet database [16] from 1955 to 2004.

The World Health Organization (WHO) estimates that up to 50 million individuals in more than 100 tropical and sub-tropical countries are infected with the mosquito-borne dengue virus (DENV) each year resulting in 500,000 hospitalizations [3,4]. With improvements in disease identification, reporting and surveillance, the number of reported dengue cases has been increasing in recent decades (Figure 1), as has the geographic range of the virus and its main vector *Aedes aegypti*, making dengue a growing public health concern, especially in developing nations. Dengue infections can result in a wide spectrum of disease severity ranging from sub-clinical to dengue fever (DF), an influenza-like illness that is commonly self-limiting, to the life-threatening dengue haemorrhagic fever (DHF)/dengue shock syndrome (DSS).

Host factors such as a previous infection with a heterologous DENV serotype, and virulence appear to play a role in determining disease severity in individuals [5-8]. Environmental factors like vector density, rainfall and temperature may affect the severity of DHF outbreaks [9]. Dengue viruses can be classified into 4 serotypes (DENV-1 to DENV-4) which have a mean nucleotide identity of 70% between the serotypes and 95% within the serotypes.

The number of DENV sequences available in the public sequence repositories has been growing steadily and the value of these sequences would be enhanced if exploratory analysis tools for performing preliminary phylogenetic analysis and search for epidemiological, geographic, and medical information were integrated with the database and convenient interactive visualization was provided. DengueInfo [10] was developed by NITD as a resource for retrieving whole genomes and associated metadata. Similarly, whole genome sequences generated at the Broad Institute can be accessed and queried directly from the institute's online database [11]. However, neither of these resources provide an integrated interface to analysis and visualization tools nor do they provide access to all dengue sequences irrespective of origin or length. To meet these needs, we extended the functionality developed by the authors of the NCBI Influenza Virus Resource to the non-segmented dengue virus. Since the DENV genome is more than 4 times larger than the largest individual influenza virus segment, multiple sequence alignments could not be calculated on request as is done for influenza virus and are instead pre-calculated offline. The alignment calculation is a three step procedure that first generates multiple protein alignments for the polyproteins derived from complete genome records of each DENV serotype, merges the serotype-specific protein alignments, and then iteratively adds shorter protein sequences. Coding sequence alignments are calculated on demand from the protein alignments. The new NCBI Virus Variation Resource is a flexible tool that can be extended to other viruses, for example West Nile virus.

## Construction and content

### Data sources and curation

The current Virus Variation Resource includes dengue and influenza virus sequences. The NCBI Influenza Virus Resource was described elsewhere [1,2]. Here we describe the extension of this resource to include dengue virus sequence data. Since the dengue genome is not segmented but more than 4 times longer than the longest influenza segment, a different approach to calculating multiple alignments is used for dengue sequences. While alignments in the Influenza Resource are calculated on demand, dengue alignments are pre-calculated to increase responsiveness and reduce server loads. Details of this approach are described in a later section.

All DENV nucleotide and protein sequences available in the public DDBJ/EMBL/GenBank repositories are evaluated for inclusion in the database. Patent sequences and sequences that contain obvious errors or vector sequences are excluded and the serotype classification is verified by comparison with a reference sequence set. Metadata (disease severity, collection date, collection location, serotype, genome region) are taken from the records, if available, or obtained from the literature. The region of the DENV genome covered by the sequence is determined by alignment and made available for queries. Newly public sequences are detected in the NCBI data stream daily and are usually added to the database within a week of becoming available.

### Data overview

**Figure 2:**
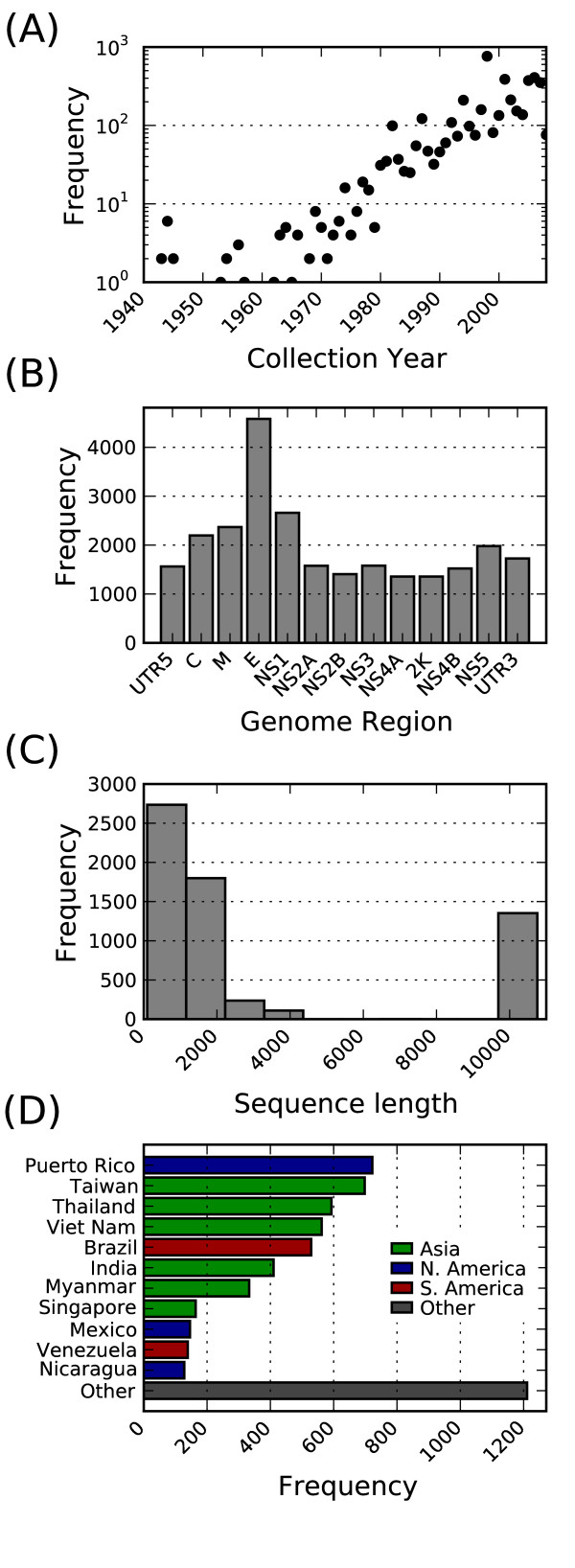
**Data overview**. Frequency of (A) collection years (N = 4543), (B) genome regions (N = 6235), (C) sequence lengths (N = 6235), and (D) collection countries (N = 5635) for dengue records in VVR.

Currently there are 6235 DENV records available in the VVR and the available metadata are summarized in Table 1. The number of sequence records available increases roughly exponentially with the year of collection (Figure 2A). The most sequenced region of the dengue genome is E and the majority of sequences are short (< 500 nt), however, there is a growing number of complete genomes available (Figure 2B, C), in large part due to the active effort to collect world-wide genome sequences. As expected, three of the top 5 most frequently represented countries in the VVR database are Asian (Taiwan, Thailand, and Viet Nam). The others are North and South American, respectively (Puerto Rico and Brazil; see Figure 2D).

**Table 1:** Data overview.

Data overview	
Total dengue records	6235
known collection country	5635 (90%)
known collection year	4543 (73%)
known disease severity	1604 (26%)
Serotypes	
DENV-1	1717 (28%)
DENV-2	2000 (32%)
DENV-3	1870 (30%)
DENV-4	648 (20%)

Overview of the characteristics of dengue records available in VVR

### Database construction

Virus Variation Resource data are stored in the relational database system MSSQL Server 2005 using a simple schema that stores nucleic acid sequences and their metadata in one table and protein sequences in a second table linked to their encoding sequences through an id field.

### Alignment construction

Multiple alignments of the available DENV protein sequences in VVR are pre-calculated offline using the following three step procedure. First, all complete protein sequences of each serotype are aligned separately in a multiple alignment step. Then, the individual intra-type alignments are merged to create a seed alignment covering the complete dengue polyprotein. Finally, incomplete sequences are aligned one by one against the sequences of the same type from the seed alignment using sequence to profile alignments. If a gap column is inserted into the profile during one of the iterative alignment steps, it is introduced into the complete seed alignment of all types to preserve consistency. When new sequences are added to the VVR database, they are added to the existing alignment through the last step of the alignment procedure. Periodically, the alignment is completely recalculated to take advantage of the increases in the number of complete sequences. Alignments are calculated with MUSCLE [12] driven by a set of custom Perl programs which rely on the BioPerl toolkit [13]. Nucleotide alignments of the coding regions are generated dynamically as codon alignments based on the protein alignments.

### Web interface and analysis tool construction

The web interface is implemented using the NCBI C++ toolkit [14] and JavaScript. The JavaScript modules were adaptated from the NCBI Influenza Virus Resource and were described previously [1,2]. C++ tools of the Influenza Virus Resource were extended to allow the use of pre-calculated dengue alignments.

## Utility and discussion

### Database query interface

**Figure 3:**
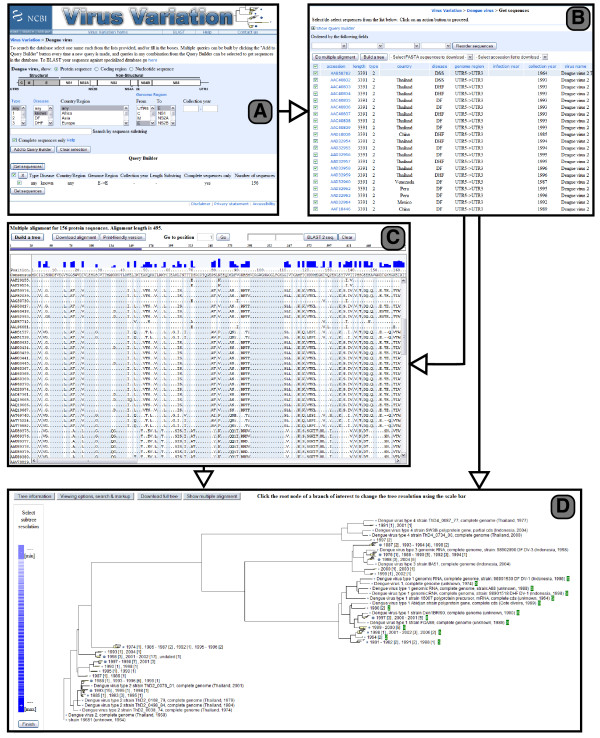
**Interface**. (A) Dengue virus query form; (B) Results page for query; (C) Multiple alignment view for results; (D) Neighbor joining tree based on nucleotide distances of codon-aligned open reading frames. Dengue serotype 1 sequences are tagged with green markers. Large branches are aggregated.

Figure 3A shows the basic query interface to the dengue virus database. Users may either search for protein sequences, their coding regions (CDS), or genomic nucleotide sequences. Additional searchable fields are: serotype (1 – 4), disease severity (DF, DHF, DSS), country or region of isolation (e.g. Europe, Puerto Rico), isolation year or year range, the genome regions included in the sequence (e.g. C, M, E), or a substring of the sequence (e.g. MNNQRKKAKN). Results may be restricted to complete sequences. Each time a query is executed by clicking "Add to Query Builder", a summary of the query parameters and the number of results are shown in the Query Builder table. An arbitrary number of queries can be executed and results for any subset of the queries can be obtained by selecting them and clicking "Get sequences", which will display the result view as seen in Figure 3B. Results can be ordered by up to three fields and a subset may be selected. The nucleotide, protein, or CDS sequence of the selected results can be downloaded in FASTA format. Alternatively, accession lists can be obtained as well.

### Multiple alignment viewer

The multiple alignment viewer is accessible from the results view. It assembles the requested pre-aligned sequences and displays them with a measure of sequence variability and a consensus anchor sequence at the top (Figure 3C). Any of the sequences can be chosen to replace the consensus as the anchor. Sequences can be selected for pairwise Blast-2-sequences alignment and aligned sequences can be downloaded in FASTA or a print-friendly format. CDS alignment are calculated dynamically based on the pre-calculated protein alignment by mapping codons to their corresponding amino acids, with coding changes highlighted in a different color. Note that only the regions selected in the query are displayed in the alignment and that the number of displayed residues in the alignment is limited to avoid delivering excessive amounts of data to client browsers. Currently the limit is 100,000 residues (for example 200 sequences of length 500), but planned improvements to the alignment viewer will likely raise this limit.

### Tree builder and viewer

Phylogenetic or clustering trees can be calculated and displayed for protein sequences or their corresponding CDS sequences. The tree builder is accessible from the results and the alignment views with the "Build a tree" button and allows sequences to be selected for inclusion based on a trade off between total length of the alignment and the exclusion of short sequences. Various measures of distance for protein and nucleotide sequences are available and are identical to those described for the NCBI Influenza Virus Resource [1]. Trees can be constructed from the distance matrices using the neighbor-joining, average linkage, complete linkage, or single linkage algorithms. To facilitate the display of trees with many leaf nodes an adaptive resolution technique in which some branches are displayed in a sub-scale representation is employed [2] (Figure 3D). Users can interactively manipulate the aggregation or refinement of any branch in the tree. In addition, certain metadata, such as year or country of isolation, can be displayed on the tree and are shown as aggregate measures for aggregated branches.

### Case study

It was reported that strains of DENV-3 circulating in Thailand prior to 1992 are distinct from those circulating after 1992, and this finding has been interpreted as an extinction of existing DENV-3 strains and the emergence of new, locally evolved strains. This event reportedly happened coincidentally with the replacement of DENV-2 with DENV-3 as the majority serotype in Thailand [15]. We demonstrate a preliminary analysis of dengue sequences using the tools of the Virus Variation Resource that supports this observation.

**Figure 4:**
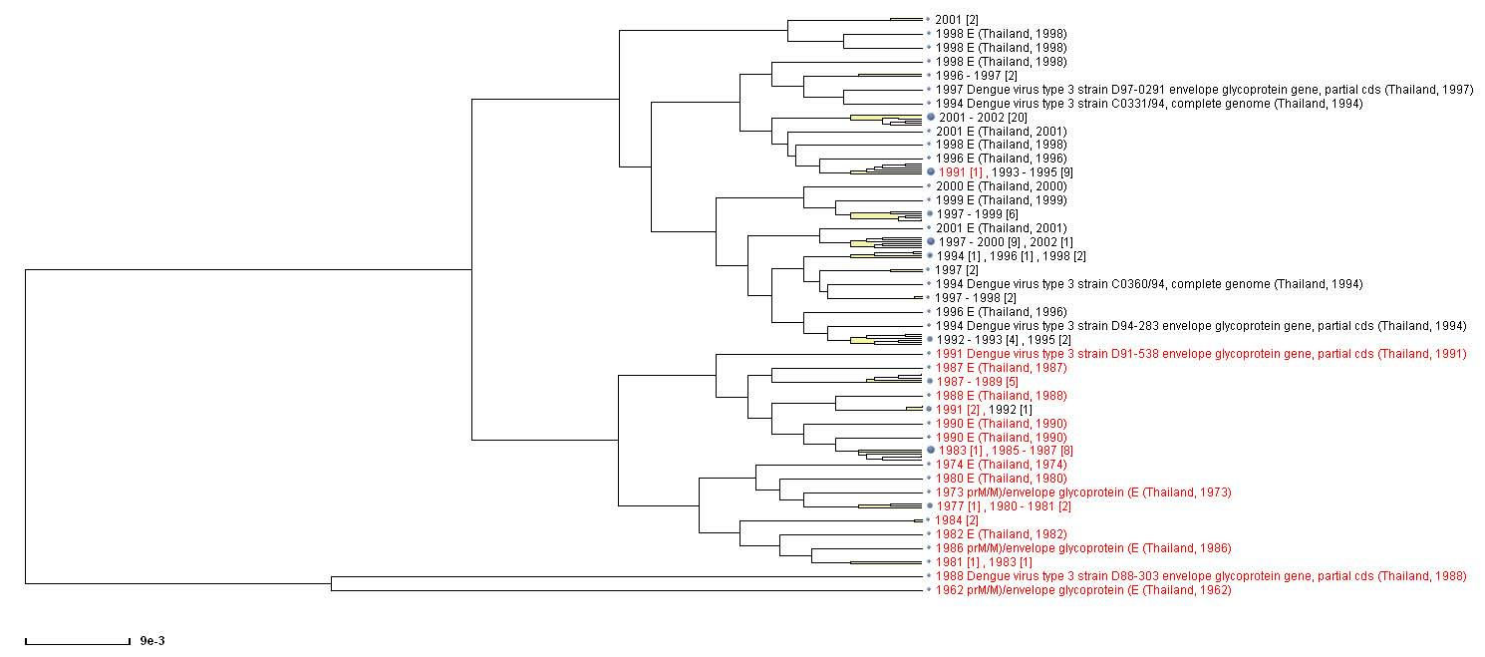
**Case study**. A clustering tree built using the the complete linkage hierarchical clustering algorithm and the F84 distances for 114 coding sequences of DENV-3 virus envelope proteins of isolates collected in Thailand and having collection year on record. Sequences obtained prior to 1992 were selected using the tree viewing option menu and highlighted in red. Most of pre-1992 DEV-3 sequences in Thailand fall in a distinct cluster.

There are 142 DEV-3 envelope protein sequences from Thailand in the database. Of those, 114 sequences have collection year on record (these can be selected by selecting collection year from 1900 to 2010). All selected sequences have complete coding sequences for envelope proteins. We selected complete linkage clustering algorithm and Felsenstein's F84 distance. The clustering tree is shown in Figure 4. Using "Viewing options, search and markup" in the tree viewer, sequences isolated before 1992 were highlighted in red. The majority of the pre-1992 sequences (92%) stay in one cluster.

### Future improvements

The Virus Variation Resource currently covers dengue and influenza viruses. However, the framework of this resource may be applied to other viruses. The Influenza Virus Resource has been very successful since its inception and we hope that additional resources in a similar mold will prove useful for other communities.

## Conclusion

Virus Variation Resources constitute a tool that allows the included virus sequences to be queried by available metadata which include geographic and medical information. Sequences resulting from these searches can then be downloaded in aligned or unaligned forms and optionally subjected to exploratory data analysis using the built-in tools. The technology for pre-calculating multiple sequence alignments can be applied to other collections, including the existing Influenza Virus Resource and a resource for the West Nile Virus that we plan to develop in the future.

## Availability and requirements

VVR databases and tools are provided as a free service by the National Center for Biotechnology Information and can be accessed at http://www.ncbi.nlm.nih.gov/genomes/VirusVariation/.

## Abbreviations

NCBI: National Center for Biotechnology Information; NIAID: National Institute of Allergy and Infectious Disease; NITD: Novartis Institute for Tropical Diseases; VVR: Virus Variation Resource; DENV: dengue virus; DF: dengue fever; DHF: dengue haemorrhagic fever; DSS: dengue shock syndrome; WNV: West Nile virus; WHO: World Health Organization; INSD: International Nucleotide Sequence Database.

## Authors' contributions

WR wrote the manuscript, curated DENV sequences, contributed to internal workflow design and implementation and was involved in overall resource design and development. LZ developed and implemented the analysis tools and their interfaces as well as the pre-alignment calculation. BK implemented the database schema and query interface to the database. TAT, MR and YB contributed to resource design and manuscript. TAT is the technical lead for the NCBI Virus Variation Resource project. All authors read and approved the manuscript.
